# Comparative Evaluation of HMG Family Proteins and miR-106a-5p in Low-Grade Non-Invasive and High-Grade Muscle-Invasive Papillary Urothelial Carcinoma

**DOI:** 10.3390/ijms27052089

**Published:** 2026-02-24

**Authors:** Natalia Domian, Magdalena Smereczańska, Małgorzata Mrugacz, Grzegorz Młynarczyk, Irena Kasacka

**Affiliations:** 1 Department of Histology and Cytophysiology, Medical University of Białystok, 15-089 Białystok, Poland; natalia.domian@umb.edu.pl (N.D.); magdalena.smereczanska@umb.edu.pl (M.S.); 2 Laboratory of Eye Rehabilitation, Medical University of Białystok, 15-089 Białystok, Poland; malgorzata.mrugacz@umb.edu.pl; 3 Department of Urology, Medical University of Białystok, 15-089 Białystok, Poland; grzegorz.mlynarczyk@umb.edu.pl

**Keywords:** urothelial carcinoma, HMGA1, HMGA2, HMGB1, miR-106a-5p, tumor grading, bladder cancer

## Abstract

Urothelial carcinoma (UC) of the bladder exhibits low- and high-grade papillary forms with distinct prognoses. High mobility group proteins (HMGA1, HMGA2, HMGB1) and miR-106a-5p are involved in tumor progression, but their interplay in UC remains incompletely understood. The aim of this study was to compare the expression of these parameters in low- and high-grade papillary UC. Tissue samples from 80 patients (40 low-grade and 40 high-grade) undergoing transurethral resection or cystectomy were analyzed, with control samples consisting of tumor-adjacent tissues without histopathological alterations obtained from the same patients. HMGA1, HMGA2, and HMGB1 protein expression was assessed immunohistochemically. Gene expression was quantified by real-time PCR, and miR-106a-5p levels were measured by droplet digital PCR. Statistical analysis was conducted using Statistica 13.3, applying one-way ANOVA with Tukey’s post hoc test and correlation analysis, with *p* < 0.05 considered significant. Expression of HMGA1 and HMGB1 was reduced in low-grade papillary urothelial carcinoma compared to control tissues, whereas both proteins were significantly increased in high-grade lesions. HMGA2 expression was minimal in low-grade tumors but partially restored in high-grade tumors. Analysis revealed the highest levels of miR-106a-5p in normal urothelium, slightly decreased in low-grade tumors, and significantly reduced in high-grade cancers. HMG proteins and miR-106a-5p demonstrate distinct expression patterns in low- versus high-grade papillary UC, which correlates with tumor aggressiveness. These molecules may serve as diagnostic and prognostic biomarkers. Their potential as therapeutic targets requires further mechanistic and translational investigation.

## 1. Introduction

Urothelial carcinoma (UC) of the urinary bladder is a heterogeneous group of malignant tumors classified based on histopathological features into two major categories: low-grade and high-grade tumors. Grading is a key parameter for both prognosis and therapy guidance in this group of cancers. The pathogenesis of UC involves complex molecular mechanisms in which aberrant gene expression and alterations in cell regulatory proteins play a key role in tumor progression and metastasis [1,2,3].

Bladder cancer remains a major public health problem worldwide, with urothelial carcinoma accounting for approximately 90% of all bladder tumors. The high rate of relapse and risk of progression justify numerous attempts to develop an optimal, effective, and reproducible classification system for grading papillary urothelial tumors. The identification of well-validated molecular markers with prognostic and predictive properties remains a crucial step. Molecular classification of UC, integrating genomic, transcriptomic, and proteomic data, is becoming increasingly important, enabling a better understanding of tumor heterogeneity and guiding the development of targeted therapies [3,4]. In this context, investigating gene expression profiles, including chromatin-associated proteins and regulatory microRNAs, may provide valuable insights into the molecular mechanisms underlying tumor grade and tumor progression in urothelial carcinoma.

High mobility group (HMG) proteins, specifically HMGA1, HMGA2, and HMGB1, are non-histone chromatin-binding proteins involved in various cellular processes, including DNA repair, transcriptional regulation, and chromatin remodeling [5,6]. Recent studies suggest that changes in the expression of these proteins may influence the invasiveness, immunity, apoptosis, and metastatic potential of malignant tumors [7,8,9]. In urothelial carcinoma, the expression of HMGA1, HMGA2, and HMGB1 is associated with tumor aggressiveness and poor prognosis for patient [10,11,12].

In addition to HMG proteins, microRNAs (miRNAs) also play a crucial role in regulating gene expression in cancer. Thus, miR-106a-5p is associated with the regulation of key genes involved in cell cycle progression, apoptosis, and tumor metastasis [13,14]. Elevated levels of miR-106a-5p in prostate and ovarian cancer have been shown to lead to reduced expression of tumor suppressors (TIMP2, KLF6), thereby facilitating cell invasion and distant metastasis [13,15]. In breast cancer, miR-106a-5p promotes tumor progression by increasing cell resistance to oxidative stress-induced death via activation of the STAT3 pathway [16]. These results suggest that miR-106a-5p plays a key oncogenic role in many types of cancers by inhibiting the expression of tumor suppressor genes and modulating key signaling pathways.

This makes miR-106a-5p a promising candidate for cancer diagnostics and therapy. However, the limited number of studies exploring the role of miR-106a-5p in modulating HMG proteins and its impact on urothelial cancer progression highlights a significant gap in current knowledge, emphasizing the need for further in-depth investigation.

The aim of this study is to perform a comparative immunohistochemical evaluation of HMGA1, HMGA2, and HMGB1 proteins, along with miR-106a-5p analysis in high- and low-grade papillary urothelial carcinomas. Furthermore, gene expression analysis will be performed to better understand the molecular dynamics that influence the differential expression of these proteins and miRNAs depending on the tumor stage.

## 2. Results

### 2.1. Immunohistochemistry

#### 2.1.1. Immunohistochemical Evaluation of HMGA1 in Papillary Urothelial Carcinoma of the Bladder

**Figure 1:**
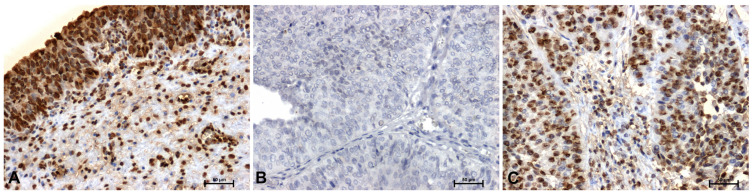
Immunoidentification of HMGA1 in papillary urothelial carcinoma: (**A**) control—urothelium from tumor-free surgical margin, showing reactive hyperplasia without dysplasia or carcinoma in situ, (**B**) low-grade, and (**C**) papillary component of muscle-invasive high-grade urothelial carcinoma (pT2). Scale bar: 50 μm.

In control tissues, strong HMGA1 immunoreactivity was observed, localized primarily in the nuclei of urothelial epithelial cells (Figure 1A). In low-grade lesions, a marked decrease in HMGA1 immunoexpression was noted (Figure 1B). In contrast, strong, particularly nuclear, immunoreactivity for this protein was observed in high-grade tumors (Figure 1C).

#### 2.1.2. Immunohistochemical Evaluation of HMGA2 in Papillary Urothelial Carcinoma of the Bladder

**Figure 2:**
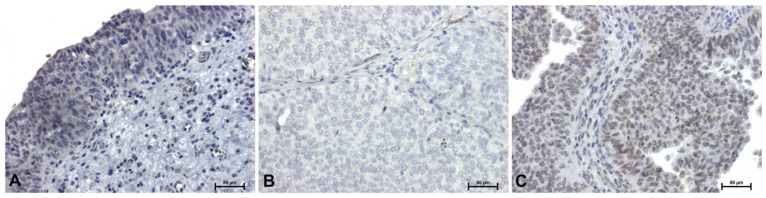
Immunoidentification of HMGA2 in papillary urothelial carcinoma: (**A**) control—urothelium from tumor-free surgical margin, (**B**) low-grade, and (**C**) papillary component of muscle-invasive high-grade urothelial carcinoma. Scale bar: 50 μm.

Very weak immunoreactivity for HMGA2 protein was demonstrated in the cytoplasm of transitional epithelial cells in control tissues (Figure 2A). In low-grade papillary urothelial carcinoma, the HMGA2 reaction was completely negative (Figure 2B). In high-grade tumors, weak but clearly visible HMGA2 immunoreactivity was observed (Figure 2C).

#### 2.1.3. Immunohistochemical Evaluation of HMGB1 in Papillary Urothelial Carcinoma of the Bladder

**Figure 3:**
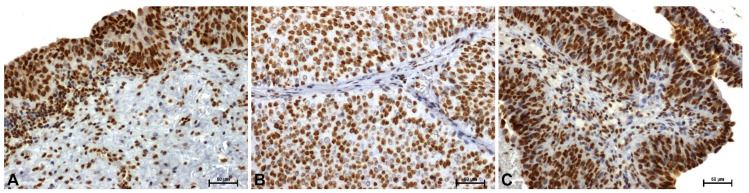
Immunoidentification of HMGB1 in papillary urothelial carcinoma: (**A**) control—urothelium from tumor-free surgical margin, (**B**) low-grade, and (**C**) papillary component of muscle-invasive high-grade urothelial carcinoma. Scale bar: 50 μm.

HMGB1 protein immunoreactivity in control tissues was very strong in the nuclei of epithelial cells and some mucosal cells (Figure 3A). The weakest HMGB1 immunoreactivity was observed in the nuclei of low-grade tumor cells (Figure 3B). High-grade lesions demonstrated strong nuclear immunoreactivity (Figure 3C).

Measurement of immunohistochemical reaction intensity using computerized image analysis, confirmed by statistical evaluation, revealed reduced immunoreactivity of HMGA1, HMGA2, and HMGB1 in low-grade papillary urothelial carcinoma compared with the control group. However, in high-grade urothelial carcinoma, the immunoreactivity of the tested proteins was highest (Table 1).

As for the relationships between the tested proteins—HMGA1, HMGA2 and HMGB1—in control tissues and urothelial papillary carcinoma, mutually positive correlations were found between each protein tested. The results of the correlation between the tested proteins are presented in Table 2.

### 2.2. Real-Time PCR

**Figure 4:**
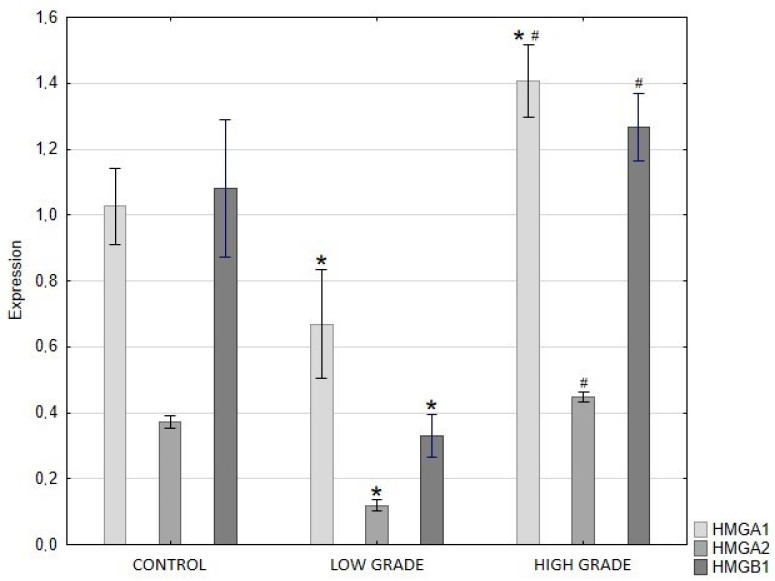
Expression levels of HMGA1, HMGA2, and HMGB1 genes in control tissue, low-grade, and high-grade papillary urothelial carcinoma, measured by real-time PCR. Data are presented as mean ± SD. Asterisks (*) indicate statistically significant differences (*p* < 0.05) compared to the control group. Hash symbols (#) indicate statistically significant differences (*p* < 0.05) between the low-grade and high-grade groups.

QRT-PCR analysis showed a significant decrease in HMGA1, HMGA2, and HMGB1 expression in low-grade papillary urothelial carcinoma compared with control tissue. In high-grade tumors, the expression of HMGA1 was markedly increased relative to the control group and was significantly higher than in the low-grade group. In contrast, HMGA2 and HMGB1 expression in high-grade tumors was not significantly different from that in the control group but was significantly higher than that in the low-grade group (Figure 4).

### 2.3. Digital PCR

**Figure 5:**
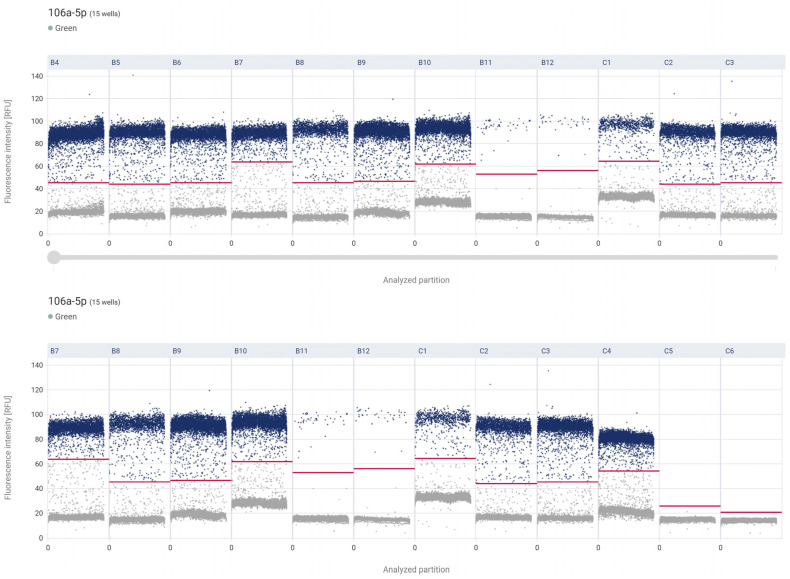
Individual sample results along the *X*-axis, with the *Y*-axis indicating the fluorescence intensity associated with miRNA 106a-5p. Each dot corresponds to the signal detected from a single reaction partition—higher intensities generally suggest a positive signal (indicating likely amplification), whereas lower intensities reflect negative or background noise. B4–B7—control, B8–B11—LG, B12–C4—HG, C5–C6—NCT.

**Figure 6:**
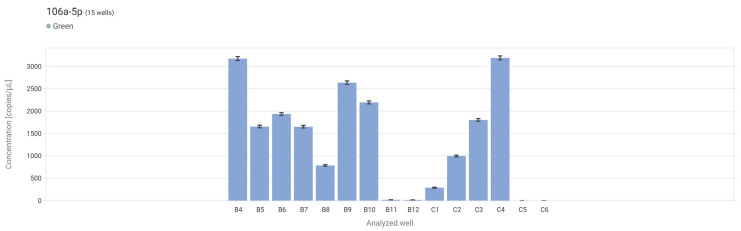
Concentration levels (copies/μL) for each sample in the form of a bar graph. B4–B7—control, B8–B11—LG, B12–C4—HG, C5–C6—NCT.

The droplet digital PCR analysis assessed miR-106a-5p expression in control tissue, low-grade, and high-grade papillary urothelial carcinoma. The results demonstrate:

The highest mean expression was observed in non-neoplastic tissue (control) and amounted to 2103.78 copies/µL. Slightly lower expression was found in low-grade urothelial carcinoma (LG) with a mean value of 1872.63 copies/µL. A marked decrease in expression was noted in high-grade carcinoma (HG), with a mean concentration of 1217.23 copies/µL. The NTC sample confirmed the assay’s specificity, with 0 copies/µL detected (Figure 5 and Figure 6).

## 3. Discussion

Bladder cancer remains a significant clinical challenge due to its high incidence and variable biological behavior. More than 90% of human bladder cancers cases are urothelial carcinomas. The term “urothelial carcinoma” is preferred over “transitional cell carcinoma”. There are two main variants of this disease, each with distinct prognoses: low-grade and high-grade tumors. Low-grade lesions are non-invasive, pose a low risk of progression, and are usually not life-threatening. High-grade tumors exhibit a more aggressive course and, without treatment, can progress to muscle-invasive lesions. Early diagnosis and staging of cancer are crucial for effective treatment. Analysis of the expression of genes and proteins associated with pathways regulating proliferation, survival, and invasiveness may improve understanding of urothelial cancer biology [17].

In this study, we performed a comparative analysis of HMGA1, HMGA2, HMGB1, and miR-106a-5p expression in low and high-grade papillary urothelial carcinoma.

Importantly, this study provides new insights into the biology of urothelial carcinoma by integrating immunohistochemical analysis and gene expression of HMGA1, HMGA2, and HMGB1 in a balanced cohort of low- and high-grade papillary tumors. To our knowledge, this is the first study to evaluate these three HMG proteins simultaneously at the mRNA and protein levels in papillary urothelial carcinoma. Moreover, this work is one of the first to assess miR-106a-5p expression in relation to HMG family expression patterns in bladder cancer.

Our results reveal that high-grade tumors are characterized by significantly increased expression of HMG proteins, along with downregulation of miR-106a-5p, which may reflect molecular mechanisms associated with tumor aggressiveness and progression.

We also demonstrated decreased HMGA1 expression in low-grade tumors compared with control tissues and increased expression in high-grade tumors. This observation is consistent with the study by Ding et al. (2014), who reported that HMGA1 is significantly upregulated in advanced bladder cancer and correlates with aggressive clinicopathological features [11]. Elevated HMGA1 levels may enhance tumor progression by modulating chromatin architecture and regulating the transcription of genes associated with cell proliferation and invasion [18,19,20].

The apparent downregulation of HMGA1 in low-grade tumors may reflect a reliance on alternative proliferative pathways in early malignant lesions. In turn, high-grade tumors appear to “reactivate” HMGA1, acting as a regulatory switch driving aggressive phenotypes. This concept echoes earlier findings by Huso et al., who described HMGA1 as a “molecular switch” required for tumor progression [21]. In turn, Khazem (2024) characterizes HMGA family proteins—particularly HMGA2—as architectural factors modulating chromatin dynamics and tumor plasticity [22].

Increased expression of HMGA1 in high-grade tumors may promote bladder cancer progression by remodeling chromatin structure and activating transcriptional programs involved in cell proliferation, migration, and invasion. HMGA1 has been described as a molecular switch for tumor progression, whose reactivation is associated with aggressive tumor phenotypes [18,19,20,21].

In the present study, HMGA2 expression showed a distinct stage-dependent pattern in papillary urothelial carcinoma. Both immunohistochemical and gene expression analyses revealed very low or undetectable HMGA2 levels in adjacent normal urothelium and in low-grade tumors. In contrast, high-grade tumors showed a significant increase in HMGA2 expression compared to low-grade lesions, but this did not exceed the levels observed in control tissue.

Our results are consistent with reports by other authors, who indicate an association of increased HMGA2 expression with invasiveness in advanced bladder cancer [11,23]. Ding et al. demonstrated elevated HMGA2 expression in bladder cancer tissues and linked it to epithelial–mesenchymal transition and aggressive tumor behavior [11]. Elevated circulating HMGA2 levels in patients with high-grade, muscle-invasive urothelial carcinoma were also demonstrated in studies by Khazem and Zetoune [23].

The reappearance of HMGA2 in high-grade tumors can be explained by the loss of certain tumor-suppressive microRNAs (miRNAs) that typically maintain its expression at low levels. One of the best-known examples is the let-7 family, which directly binds to the 3′-UTR of HMGA2 and prevents its translation; when this control is lost, HMGA2 can promote epithelial–mesenchymal transition (EMT) and tumor progression [24]. In bladder cancer, miR-485-5p has been shown to block HMGA2-driven EMT and invasion, while reduced levels of this miRNA restore HMGA2 activity and tumor aggressiveness [25]. Similar mechanisms have been reported in other cancers, for instance, the miR-302a-5p/367-3p-HMGA2 axis in endometrial carcinoma; additionally, miR-142-3p in cervical cancer, miR-219-5p and miR-154 in prostate cancer, the miR-98/HMGA2/periostin axis in laryngeal carcinoma, and several miRNAs including miR-101, miR-204-5p, miR-485-5p, and miR-150 in various tumor types [26,27].

Re-expression of HMGA2 in high-grade tumors may result from loss of tumor-suppressive microRNA-mediated regulation and contribute to epithelial–mesenchymal transition (EMT), thereby increasing tumor invasiveness and metastatic potential. Previous studies have demonstrated the involvement of HMGA2 in EMT and the aggressive behavior of advanced urothelial carcinoma and other malignancies [11,23,25,27].

Our results demonstrated a distinct stage-dependent pattern of HMGB1 expression in papillary urothelial carcinoma. High levels of HMGB1 were found in control tissues, but expression was decreased in low-grade tumors and significantly increased in high-grade lesions. These findings are consistent with literature reports indicating higher HMGB1 expression in more aggressive forms of bladder cancer. Suren et al. demonstrated significantly higher levels of HMGB1 in invasive urothelial carcinoma compared with non-invasive tumors, supporting the association between HMGB1 expression and tumor aggressiveness [28]. Furthermore, as described by other researchers, HMGB1 is a context-dependent protein and is characterized by altered expression in advanced malignancies [9,29].

Increased expression of HMGB1 observed in high-grade tumors may reflect its context-dependent role in cancer, encompassing both nuclear functions related to transcriptional regulation and extracellular signaling that promotes inflammation, invasion, and tumor progression. In urothelial carcinoma, elevated HMGB1 levels have been associated with more aggressive and invasive tumor phenotypes [9,28,29].

In the examined control tissues and in low- and high-grade papillary urothelial carcinoma, positive correlations were found between the expression of HMGA1, HMGA2, and HMGB1, suggesting their interdependent regulation and potential involvement in similar biological mechanisms in both physiological and neoplastic conditions.

Our droplet digital PCR analysis revealed the highest expression of miR-106a-5p in control tissue, a slight decrease in low-grade tumors, and a significant reduction in high-grade carcinomas, suggesting a context-dependent tumor suppressive role of miR-106a-5p in urothelial carcinoma. This observation contrasts with reports from other malignancies, such as prostate, ovarian, and breast cancer, where miR-106a-5p has been shown to function predominantly as an oncogenic miRNA, highlighting the tissue context-specific nature of miRNA function [13,14,15,16,30,31]. Although the direct targeting of miR-106a-5p to HMGA2 in bladder cancer remains unconfirmed, the existing literature supports the involvement of miRNAs in the regulation of HMGA2 expression. For example, the let-7 family of miRNAs directly interacts with HMGA2 by binding to its 3′-UTR, thereby inhibiting its expression and suppressing tumor progression; disruption of this interaction can lead to increased oncogenic transformation [32]. Additionally, a comprehensive review by Hashemi et al. discusses how various miRNAs, including let-7, modulate HMGA2 expression, influencing hallmarks of cancer such as apoptosis, proliferation, EMT, invasion, and metastasis [7].

Downregulation of miR-106a-5p in high-grade tumors may indicate a context-dependent tumor-suppressive function and reflect broader dysregulation of microRNA-mediated posttranscriptional control during tumor progression. Although the direct effect of miR-106a-5p on HMGA2 in bladder cancer has not yet been confirmed, numerous evidence indicates that disruption of the miRNA network leads to increased expression of HMGA2 and promotes EMT, invasion and metastasis [7,24,32]. Importantly, the inverse expression pattern observed between miR-106a-5p and HMG family proteins may suggest a potential regulatory association; however, no functional validation was performed in this study. Therefore, this observation should be interpreted as hypothesis rather than evidence of a direct mechanistic interaction. Further studies using miR-106a-5p modulation models are needed to confirm a direct effect on HMG expression and tumor invasiveness.

Although the results of this study are interesting and highlight the potential role of the analyzed markers in the progression of urothelial carcinoma, a limitation is the relatively small sample size, which may limit the generalizability of the findings to the broader patient population. Moreover, the analysis focused primarily on expression profiles, without in-depth functional studies that could better elucidate the molecular mechanisms underlying the observed associations. Another limitation of the study is that only staining intensity was assessed, without formally determining the percentage of positive tumor cells. Future studies with larger cohorts and functional analyses are needed to confirm these findings and clarify their biological and clinical significance.

Importantly, none of the patients included in this study received neoadjuvant chemotherapy prior to surgery, minimizing potential treatment-related modulation of gene expression profiles. Nevertheless, intratumoral molecular heterogeneity is an inherent feature of urothelial carcinoma and may contribute to variability in biomarker expression. Although representative areas were selected for analysis, this aspect remains a limitation of tissue-based studies.

During bladder cancer surgery, the peritumoral margin depends on the extent of the macroscopically visible lesion. Typically, resection included the visible lesion along with a margin of at least 10–15 mm of tissue around the tumor. Histopathological examination revealed no tumor cells or visible pathological changes in the reference tissues (negative margin).

In summary, our study demonstrates distinct differences in the expression of HMGA1, HMGA2, HMGB1, and miR-106a-5p between low- and high-grade papillary urothelial carcinoma. High-grade tumors are characterized by increased expression of HMGA1 and HMGA2, along with decreased levels of miR-106a-5p, which may be associated with tumor aggressiveness and disease progression. HMGB1 exhibits a biphasic expression pattern, reflecting its dual intracellular and extracellular roles in cancer biology. Taken together, these findings suggest that HMG proteins and selected miRNAs may serve as potential biomarkers of malignancy grade and prognosis in cancer. Although HMG proteins represent attractive molecular candidates due to their role in chromatin remodeling and tumor progression, their ubiquitous expression and multifunctional nuclear roles pose significant challenges for direct pharmacological targeting. Further research is needed to better define the underlying molecular mechanisms and to explore their potential clinical applications in diagnosis and targeted therapy [33].

## 4. Materials and Methods

The study was conducted on tissue samples collected from patients after surgical removal of bladder tumors at the Department of Urology, Medical University of Bialystok. The study was approved by the Bioethics Committee of the Medical University of Bialystok, under the code APK.002.109.2023. All procedures were in accordance with applicable ethical guidelines and regulations. The research adhered to the principles outlined in the Declaration of Helsinki regarding research involving human participants.

The study included patients who undergoing transurethral resection of bladder tumors or radical cystectomy. Tissue samples for analysis were collected directly from the tumor during surgery. Samples for immunohistochemical analysis were fixed in 10% buffered formalin immediately after collection. For real-time PCR analysis, samples were placed in RNAlater solution and stored at −80 °C. The study group consisted of 40 patients diagnosed with high-grade papillary urothelial carcinoma and 40 patients with low-grade tumors (Table 3). Control material consisted of tumor-adjacent tissues, collected from the same patients.

### 4.1. Identification of HMGA1, HMGA2, and HMGB1 by Immunohistochemistry

Immunohistochemical analysis of HMGA1, HMGA2 and HMGB1 protein expression was performed on tissue sections from all patients, including both tumor samples and corresponding control urothelium from tumor-free surgical margins.

Immunohistochemical analysis was conducted following a standardized protocol. Tissue specimens were initially fixed in 10% formalin to preserve morphology. The samples were subsequently dehydrated through a graded ethanol series, cleared in xylene, and embedded in molten paraffin wax. Once embedded, the tissue blocks were allowed to solidify before being sectioned.

Using a microtome, tissue sections approximately 4 μm thick were cut, mounted onto glass slides, and dried thoroughly. The sections underwent deparaffinization and rehydration before further processing. Primary antibodies targeting HMGA1 (Abcam ab252930, 1:600 ABCAM. Discovery Drive, Cambridge Biomedical Campus, Cambridge, CB2 0AX, UK), HMGA2 (Abcam ab207301, 1:250), and HMGB1 (ab79823, 1:400; ABCAM. Discovery Drive, Cambridge Biomedical Campus, Cambridge, CB2 0AX, UK) were diluted and applied to the tissue. Antigen retrieval was performed using Target Retrieval Solution (citrate buffer, pH 6.0; S2369; Agilent Technologies, Inc. Santa Clara, CA, USA) at 125 °C. To inhibit endogenous peroxidase activity, the slides were treated with 0.3% hydrogen peroxide for 10 min.

After overnight incubation with the primary antibodies at 4 °C, sections were treated with an HRP-conjugated secondary antibody (DAKO REAL™ EnVision™ Detection System, K5007, Agilent Technologies, Inc. Santa Clara, CA, USA) for 1 h. Visualization of the antibody–antigen complexes was achieved using DAB chromogen. Hematoxylin QS (H-3404, Vector Laboratories (Newark, CA, USA)) was used for nuclear counterstaining. Each step of the staining procedure was followed by rinsing the slides in Wash Buffer (S3006 Agilent Technologies, Inc. Santa Clara, CA, USA).

Antibody specificity was verified through the use of both negative and positive controls. Negative controls were prepared by omitting the primary antibody and replacing it with either diluent or a non-specific antibody of the same isotype, species, and concentration. Positive controls utilized tissues known to express the target antigen, as recommended by the antibody manufacturer. In the positive controls, staining was confined to expected structures, while background tissues remained unstained. No specific signal was observed in the negative controls.

Immunohistochemical evaluation was based on staining intensity, as the proportion of positive tumor cells was relatively uniform across all samples. Intensity was scored as weak, moderate or strong nuclear staining. In cases of heterogeneous staining, a predominant intensity was observed in the tumor cells.

Histological evaluation was performed using an Olympus BX43 light microscope equipped with an Olympus DP12 digital camera (Olympus Corp. (Tokyo, Japan)). Digital images of both cancerous and normal bladder tissue were analyzed morphometrically using NIS Elements AR 3.10 software (Nikon). The staining intensity for each antibody was assessed using grayscale.

### 4.2. Real-Time PCR

Fresh-frozen bladder cancer specimens and matched adjacent normal tissue were collected into Eppendorf tubes containing RNA-later (Invitrogen AM7021 (Waltham, MA, USA)) and stored at −80 °C. Final classification into tumor or control groups was based on the definitive histopathological diagnosis of the corresponding surgical specimens.

Total RNA was isolated from tumor tissues and corresponding control tissues adjacent to the tumor from the same 80 patients. Only samples fulfilling quality criteria were included in further analysis.

Total RNA was extracted using the Macherey-Nagel NucleoSpin^®^ RNA kit. RNA quantity and purity (A_260_/_280_) were assessed using a NanoDrop 2000 (Thermo Scientific (Waltham, MA, USA)). First-strand cDNA was synthesized from total RNA using BIO-RAD’s iScript™ (Hercules, CA, USA) Advanced cDNA Synthesis Kit in a SureCycler 8800 thermal cycler (Agilent Technologies (Santa Clara, CA, USA)) under the following cycling conditions: 46 °C for 20 min, 95 °C for 1 min, and a final hold at 4 °C.

Quantitative real-time PCR was performed on a Stratagene Mx3005P instrument using SsoAdvanced™ Universal SYBR^®^ Green Supermix (BIO-RAD). Gene expression assays (PrimePCR™ SYBR^®^ Green) were employed with the following unique assay IDs: HMGA1 (human PrimePCR assay qHsaCED0004947 BIO-RAD), HMGA2 (human PrimePCR assay qHsaCED0021425 BIO-RAD) and HMGB1 (human PrimePCR assay qHsaCED0020471 BIO-RAD).

All reactions were performed in duplicate in 10 μL volumes with the following thermal profile: 95 °C for 2 min (enzyme activation), then 40 cycles of 95 °C for 5 s (denaturation) and 60 °C for 30 s (annealing/extension). Control reactions included no-RT controls, no-template controls, and melting curve analysis to verify single-product amplification.

Relative quantification of gene expression was performed using the ΔΔCt method. Gene expression levels were normalized to GAPDH and expressed relative to the control tissue.

### 4.3. Digital PCR

Digital PCR analysis was performed as a pilot study on a subset of 12 biological samples (four control tissues, four low-grade tumors, and four high-grade tumors). No-template controls (NCTs) were included in each run to verify assay specificity.

RNA extraction from urothelial carcinoma of the bladder tissues was performed using the miRNeasy Tissue/Cells Advanced Micro Kit (Qiagen, Copenhagen, Denmark, cat. no. 217684) following the manufacturer’s protocol. A 4 mm^3^ section was excised from each tissue specimen and placed into a 1.5 mL reaction tube with 60 μL of lysis solution containing 1% β-mercaptoethanol. Tissues were homogenized using a disposable polypropylene pestle and then lysed in 700 μL of QIAzol Lysis Reagent (Qiagen, cat. no. 79306). Subsequent steps were carried out as per the manufacturer’s instructions. RNA was eluted using 40 μL of RNase-free water (Qiagen, cat. no. 129112). Purity, concentration, and contamination of RNA samples were assessed using a NanoDrop spectrophotometer (Thermo Fisher Scientific, Waltham, MA, USA). Reverse transcription of RNA was performed using the miRCURY LNA RT Kit (Qiagen, cat. no. 339340). For fresh frozen urothelial carcinoma of the bladder tissues, RNA concentration was adjusted to 5 ng/μL per sample, with 2 μL added to a total reaction volume of 10 μL, including 0.5 μL of UniSp6 RNA spike-in. Digital PCR (dPCR) reactions utilized the miRCURY LNA miRNA PCR Assays kit (Qiagen, cat. no. 39306) in a 96-well plate format, analyzed on the QIAcuity One nucleic acid detection instrument (Qiagen) with QIAcuity Software Suite 2.1.8.20 software (Qiagen). Reaction mixtures consisted of 4 μL 3x EvaGreen PCR Master Mix (Qiagen, cat. no. 250111), 1.2 μL miRCURY LNA PCR Assay (10×) (Qiagen, cat. no. 339306), 3 μL cDNA template (Qiagen), and 3.8 μL RNase-free water (Qiagen, cat. no. 129112), totaling 12 μL. A 96-well nanoplate with 8500 partitions was used. The miRNA under study was miRNA-106a-5p (Qiagen, cat. no. YP00204563). The reaction mix was dispensed into standard PCR plates, and each well content was transferred to the QIAcuity Nanoplate (Qiagen). Cycling conditions included initial PCR heat activation for 2 min at 95 °C, followed by 40 cycles of denaturation at 95 °C for 15 s and annealing at 60 °C for 1 min, concluding with a 5 min cool-down at 40 °C. Raw data were analyzed using the QIAcuity Software Suite (Qiagen).

### 4.4. Statistical Analysis

Statistical analysis was performed using Statistica version 13.3 software. Data distribution was assessed prior to analysis. Differences between groups were evaluated using one-way analysis of variance (ANOVA), followed by Tukey’s post hoc test for multiple comparisons. A *p*-value < 0.05 was considered statistically significant.

To analyze the correlation between the tested proteins, r2, β coefficient, and statistical significance (*p*) were calculated. The relationship between two variables was acknowledged to be statistically significant at the value of the β coefficient for which *p* < 0.05.

## Figures and Tables

**Table 1:** The intensity of immunoreaction determining HMGA1, HMGA2 and HMGB1 in adjacent normal tissues, low- and high grade papillary urothelial carcinoma.

Intensity of Immunohistochemical Reaction in Low- and High Grade Papillary Urothelial Carcinoma			
Scale From 0 (White Pixel) to 256 (Black Pixel)			
	HMGA1	HMGA2	HMGB1
Adjacent normal tissues	164.75 137.63–174.81	70.35 36.65–90.93	173.89 159.69–210.31
Low-grade papillary UC	94.85 * 79.17–106.1	42.62 33.42–58.55	102.13 * 74.81–144.99
High-grade papillary UC	198.76 **, # 172.34–226.26	88.1 **, # 70.05–113.38	206.37 **, # 216.33–228.9

Data are shown as a median and minimum and maximum values for HMGA1, HMGA2 and HMGB1 in adjacent normal tissues, low- and high grade papillary urothelial carcinoma. * *p*  <  0.05—low grade UC vs. adjacent normal tissues. ** *p*  <  0.05—high grade UC vs. adjacent normal tissues. # *p*  <  0.05—high grade UC vs. low grade UC.

**Table 2:** Correlation analysis of HMGA1, HMGA2 and HMGB1 in control tissue and urothelial papillary carcinoma.

Urothelial Papillary Carcinoma			
HMGA1	HMGA2	HMGB1	
-	β = +0.829 *p* = 0.00000 * r^2^ = 0.6082	β = +1.3719 *p* = 0.0000 * r^2^ = 0.8159	HMGA1
	-	β = +1.2783 *p* = 0.0000 * r^2^ = 0.8003	HMGA2
		-	HMGB1

Data shown as β coefficient, r^2^ and statistical significance, where * *p* < 0.05.

**Table 3:** Patient characteristics/bladder cancer.

	Low-Grade Bladder Cancer (Non-Muscle Invasive)	High-Grade Bladder Cancer (Muscle Invasive)
Average age	60.8	66.2
♀	26	12
♂	14	28
Previous treatment > neoadjuvant chemotherapy	0	0
Staging	pTaN0M0—all patients	pT2N0M0—all patients
Average BMI	27.16	29.84
Metastasis	0	0
Arterial hypertension	10	12
Diabetes type 2	5	4
Atrial fibrillation	2	1
Hyperthyroidism	2	3

## Data Availability

The data presented in this study are available on request from the corresponding author. The data are not publicly available due to privacy.
